# Relaxation damping in oscillating contacts

**DOI:** 10.1038/srep16189

**Published:** 2015-11-09

**Authors:** M. Popov, V.L. Popov, R. Pohrt

**Affiliations:** 1 Berlin University of Technology, 10623 Berlin, Germany; 2 National Research Tomsk State University, 634050 Tomsk, Russia; 3 National Research Tomsk Polytechnic University, 634050 Tomsk, Russia

## Abstract

If a contact of two purely elastic bodies with no sliding (infinite coefficient of friction) is subjected to superimposed oscillations in the normal and tangential directions, then a specific damping appears, that is not dependent on friction or dissipation in the material. We call this effect “relaxation damping”. The rate of energy dissipation due to relaxation damping is calculated in a closed analytic form for arbitrary axially-symmetric contacts. In the case of equal frequency of normal and tangential oscillations, the dissipated energy per cycle is proportional to the square of the amplitude of tangential oscillation and to the absolute value of the amplitude of normal oscillation, and is dependent on the phase shift between both oscillations. In the case of low frequency tangential oscillations with superimposed high frequency normal oscillations, the dissipation is proportional to the ratio of the frequencies. Generalization of the results for macroscopically planar, randomly rough surfaces as well as for the case of finite friction is discussed.

It is well known that oscillating tangential contacts exhibit frictional damping due to slip in parts of the contact. Solutions for this behavior in the case of spherical surfaces were given by Mindlin *et al.*^1^ in 1952. This contact damping plays an important role in numerous applications in structural mechanics^2^, tribology^3^ and materials science^4^. Since this damping arises due to partial slip in the contact of bodies with curved surfaces, when the coefficient of friction tends towards infinity, slip disappears, frictional losses are eliminated, and the oscillation damping becomes zero^1^. However, when a contact oscillates in *both normal and tangential* directions, there is another, purely elastic loss mode that we refer to as “relaxation damping”. To our knowledge this phenomenon has not yet been discussed in the literature. Damping due to a combination of normal and tangential oscillations has been studied recently by Davies *et al.*^5^ for smooth two-dimensional profiles and by Putignano *et al.*^6^ for rough surfaces. However, the fact that dissipation exists even in the limiting case of an infinite coefficient of friction, when relative frictional movement of contacting bodies does not occur, went unnoticed. This effect is an example of purely “non-dissipative” damping, like the Landau damping in a collisionless plasma^7^.

In its essence the proposed loss mechanism is similar to a spring that is deflected and abruptly released, converting the stored energy into elastic waves that are eventually dissipated. If we consider a body that is pressed into a plane, then moved tangentially (with “stick” conditions in the contact), and finally lifted in the normal direction, the accumulated shear energy will eventually be lost even if there is no slip in the contact area and the material is purely elastic. Thus, an apparently non-dissipative system shows dissipation. The same will also happen in contacts that oscillate normally and tangentially at the same time, even if the motion is very slow (quasi-static.) At first glance it seems contradictory that a slowly moving, non-dissipative system shows dissipation. The physical reason for this dissipation is the infinite stress concentration at the borders of a tangential contact. Due to the stress singularity, infinitely rapid movements occur in the material even in the case of quasi-static macroscopic movement of the contacting bodies, similar to the dissipation from elastic instabilities in the Prandtl-Tomlinson-model^8^,^9^,^10^. The physical nature of relaxation damping can be understood and analyzed very simply in the framework of the method of dimensionality reduction (MDR). For small oscillation amplitudes, the dissipation rate can be calculated analytically.

## Results

### Preliminary remarks

**Figure 1:**
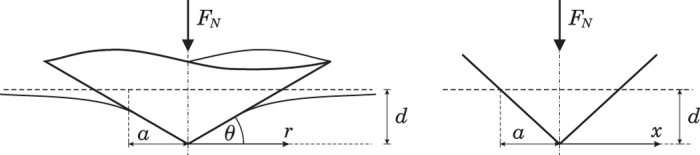
(**a**) Contact of a cone with a half-space and (**b**) the corresponding MDR-transformed one-dimensional profile.

Consider a contact between two axially-symmetric elastic bodies with moduli of elasticity of 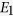 and 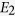, Poisson’s numbers of 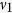 and 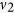, and shear moduli of 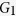 and 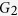, accordingly. We denote the difference between the profiles of the bodies as 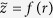, where 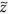 is the coordinate normal to the contact plane, and 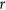 is the in-plane polar radius. The profiles are brought into contact and are subjected to a superposition of normal and tangential oscillation with small amplitudes. This contact problem can be reduced to the contact of a rigid profile 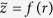 with an elastic half-space, Fig. 1a.

In our analysis we use the method of dimensionality reduction, MDR^11^. MDR is based on the solutions for the normal contact by Galin^12^ and Sneddon^13^ as well as their extensions for tangential contacts by Cattaneo^14^, Mindlin^15^, Jäger^16^ and Ciavarella^17^. In the framework of the MDR, two preliminary steps are performed^11^: First, the three-dimensional elastic half-space is replaced by a one-dimensional linearly elastic foundation consisting of an array of independent springs, with a sufficiently small separation distance 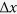 and normal and tangential stiffness 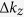 and 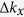 defined according to the rules


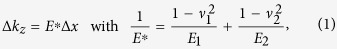



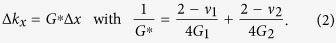


In the second step, the three-dimensional profile 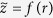 is transformed into a one-dimensional profile according to


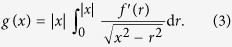


If the MDR-transformed profile 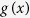 is indented into the elastic foundation and is moved normally and tangentially according to an arbitrary law, the contact radius and the force-displacement relations of the one-dimensional system will exactly reproduce those of the initial three-dimensional contact problem (proofs have been done in^18^ and^11^). The MDR solution is as accurate as the solutions of Cattaneo^14^ and Mindlin^1^: the solution contains an inaccuracy, which has been shown to be generally quite small^19^. From the correctness of the force-displacement relations, it follows that the work and the dissipated energy will be reproduced correctly as well.

In the following, we consider, without loss of generality, a rigid conical indenter


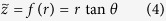


in contact with a half-space, Fig. 1a.

The one-dimensional MDR image of the conical profile (4), according to (3), is


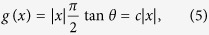


where 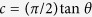 is the slope of the one-dimensional equivalent profile, Fig. 1b. The generalization for an arbitrary axis-symmetrical shape can be made very easily: if the amplitude of normal oscillation is sufficiently small compared to the indentation depth of the indenter, the shape of the edge of the contact will always be approximately linear. For determining the energy dissipated during one cycle of oscillation, only the zone near the edge of contact (of one-dimensional MDR model) must be considered because dissipation can only take place where the surfaces come in and out of contact. In this case, all axially-symmetric indenters will behave like conical indenters and the slope 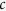 at the edge of the contact of the one-dimensional MDR-transformed profile will be the only shape-related parameter. For example, for a parabolic indenter 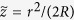, the MDR-transformed profile is 
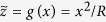
 and the edge slope is 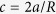 where 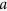 is the contact radius. The parameter 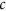 can also be represented in a universal form that does not depend on the profile shape: The incremental contact stiffness is known to be equal to 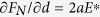, see [20]. Deriving this equation once more gives 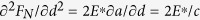. Thus, the slope of the MDR-transformed profile can be calculated as


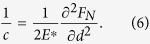


In the following, we consider energy dissipation in two cases: (a) oscillations in the normal and tangential direction with equal frequencies, (b) oscillation in the normal direction with much higher frequency than in the tangential direction.

### Normal and tangential oscillations with equal frequencies

**Figure 2:**
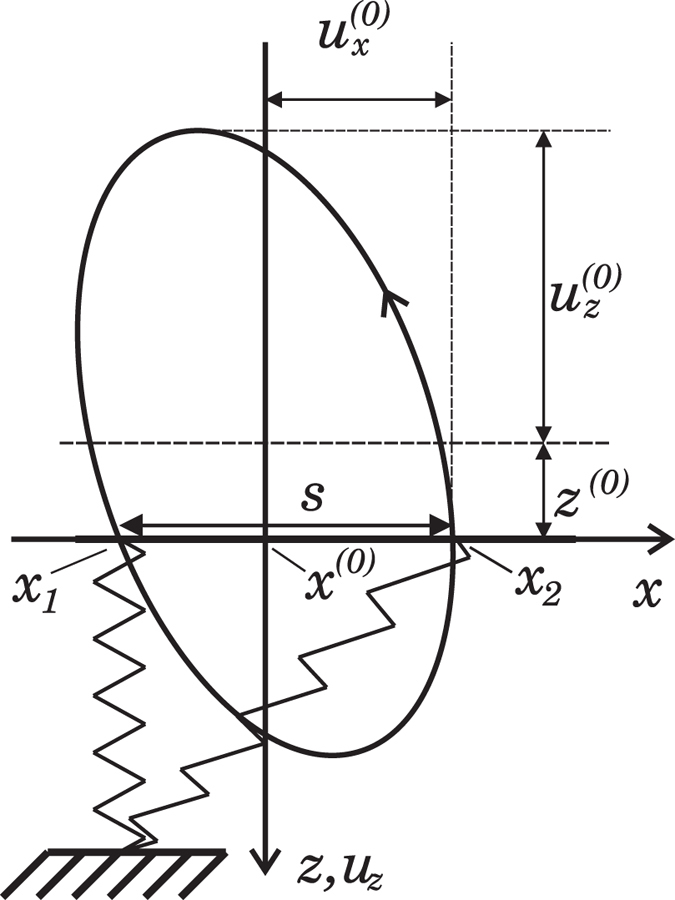
A point of the rigid surface with the initial coordinate z=−z^(0)^ oscillates around this position. It comes into contact with a spring in point 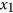 and loses contact in point 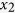.

Let the profile oscillate harmonically with a normal amplitude 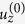, a tangential amplitude 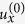 and a phase difference 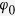. To study the effect of relaxation damping in the pure, we assume an infinite friction coefficient between both bodies. Since the springs of elastic foundation in the MDR model are independent, it is sufficient to analyze the energy dissipation of a single spring (Fig. 2), and then to sum over all springs which come into contact during an oscillation cycle. Consider a point of the rigid indenter with the initial coordinates 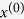, 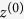. Its coordinates during the oscillatory motion can be written as 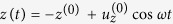 and 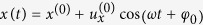. If 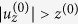, the point of the rigid surface will come into contact with one of the springs of the elastic foundation in point 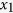 and will drag it along to point 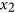, where contact is lost and the spring relaxes over the distance 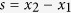. The coordinates 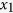 and 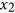 are determined by setting 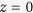. After simple calculations we get


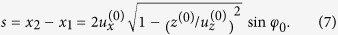


The energy dissipated by a single spring during one cycle is equal to the energy stored in the stressed spring at the time of its release:


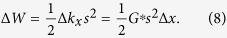


Energy dissipation occurs only if the point of the surface was in contact with the substrate during only a part of the cycle. This is the case for all points which satisfy the condition


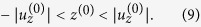


Substituting 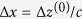 in (8) and integrating over the interval (9), we obtain the total dissipated energy per cycle:


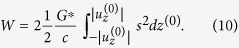


The factor “2” takes into account that there are two symmetric regions on both sides of the contact giving equal contributions to dissipation (this complete symmetry is only valid in the standard half-space-approximation used in this paper). Substitution of (7) into (10) and evaluation of the integral finally gives


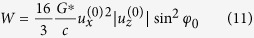


or in the shape invariant form, using (6),


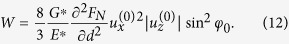


### Low frequency tangential oscillation with high frequency normal oscillation

If the frequency of normal oscillation 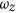 is much larger than 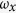, the frequency of tangential oscillation, the body will move tangentially with an approximately constant velocity 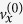 during any given cycle of normal oscillation. Let the 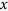-coordinate of a point of the indenter be 
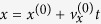
, while the 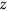-coordinate is defined by 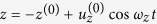 as before. The times at which a spring is coming into contact with the indenter (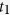) and is released (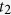) are given by the condition 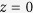, from which it follows that 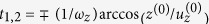. For the distance 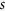, we get the following result:


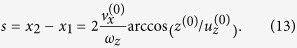


Substituting into (10) and evaluating the integral, we get the energy dissipated per normal oscillation cycle:


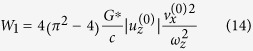


from which we obtain the average dissipated power in a normal oscillation cycle:


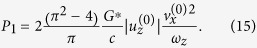


By defining 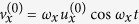 and integrating over one cycle of tangential oscillation (from 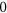 to 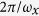) we find the dissipated energy to be:


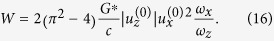


In the shape-invariant form, the energy dissipation per cycle of tangential oscillation is:


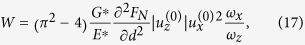


which is nearly identical to (12), save for the different constant and a dependence on the ratio of frequencies, instead of the phase difference. As stated before, this result is only valid if 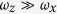.

### Further generalization

We would like to stress that in spite of the fact that the relaxation losses (11)–(12) and (16)–(17) have been derived in a one-dimensional model, they represent, due to the MDR theorems, the exact three-dimensional results for axis-symmetric profiles. In the shape invariant form (12) and (17) they are even applicable to multi-contact systems with independent contacts, as e.g. represented by the Greenwood and Williamson model of contact of rough surfaces^21^. This follows directly from the linearity of the energy losses with respect to the normal force. The shape invariance of the results (12) and (17) suggests that these may even be exact relations applicable to any three-dimensional contact topography^*^.

To illustrate this universality and to provide additional numerical validation of the general equations (12) and (17), we carried out a series of three-dimensional, full-Cerruti-type numerical simulations of oscillating contacts using the methods described in detail in^23^ and^25^, with some modifications. We assumed that normal and tangential deformation are uncoupled i.e. tangential stresses do not alter the normal contact solution. This is strictly valid only when both materials are identical, or one is incompressible and the other one is either incompressible or perfectly rigid. In order to handle the case of infinite friction, the boundary conditions were altered to force all contact points into an individual horizontal deformation depending on their time of entering into contact.

**Figure 3:**
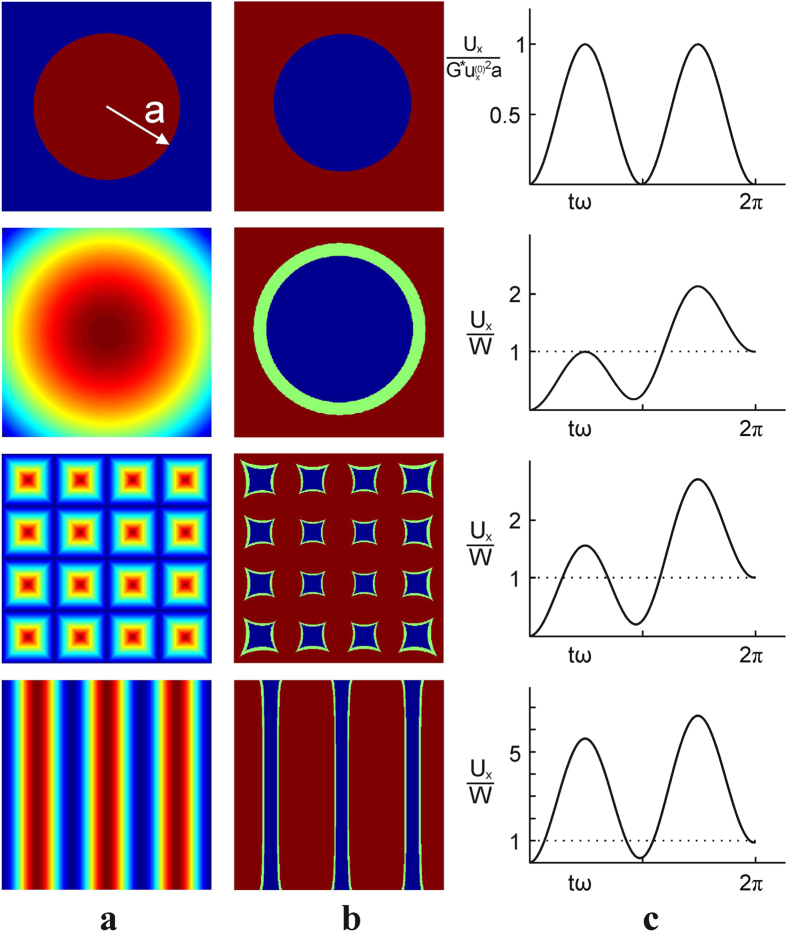
(**a**) Various surface profiles used to validate Eq. (12) by direct three-dimensional simulations of oscillating contact: a sharp-edged cylindrical profile; a parabolic surface; an arrangement of 16 pyramid indenters; a series of elongated sinusoidal profiles. (**b**) The contact configurations for the corresponding profiles. The minimum contact regions of a complete cycle are colored in blue and the additional regions at maximum contact in green. (**c**) Time plots of the work done by external forces in the x-direction on the system over one period of oscillation, normalized by the prediction *W* according to eq. (12). In the first example, the contact area is not changed in the cycle so no dissipation takes place. In the other cases, the curves reach unity after one cycle, thereby confirming the validity of eq. (12). In all studied cases, the direct simulations reproduce the analytical result with an error not exceeding 5%, which is primarily caused by the difficulty of determining 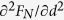 from discrete samples.

The essential findings related to these simulations are summarized in Fig. 3: For 4 different surface topographies (left column), the minimum and maximum contact areas are shown (middle column) as well as the time plots of the work done by the external force in the 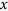-direction (right column). The total work done during one period (values reached at 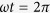) is the dissipated energy. The horizontal dotted line shows the unity-normalization according to Eq. (12). One can see that the three-dimensional results coincide with the analytical prediction not only for axis-symmetrical profiles, but also for profiles having an “arbitrary” different form. We thus can conclude that Eq. (12) can be universally applied to contacts of arbitrarily shaped bodies. The same will be valid of course for Eq. (17).

Let us apply Eq. (12) to an important class of nominally flat rough surfaces (surfaces having a long wavelength cut-off of the power spectrum of roughness) in contact with a flat counterpart. For such surfaces, the relation between the normal force and the indentation depth is known to be 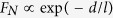
11,22, where 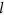 is of the order of magnitude of the rms roughness. For the second derivative of the force, we have 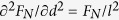. Thus, for rough surfaces, the damping is proportional to the normal force. Substitution into (12) gives:


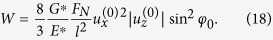


### Physical interpretation

Finally, let us come back to the physical nature of the relaxation damping. Brillouin was probably the first to recognize that a non-vanishing dissipation at low velocity can only occur if there are some discontinuous jumps from one state to another in the system^26^. In other words, movement with finite velocity must occur in the system even if it is driven quasi-statically. Such rapid movements due to elastic instabilities are e.g. the reason for the appearance of finite dissipation in the celebrated Prandtl-Tomlinson-model^8^. At first glance, the oscillating contacts discussed in this Paper do not lead to any rapid movements. However, a singularity of stresses does exist at the border of the contact. This singularity leads to infinitely rapid movements even if the configuration of the contact changes quasi-statically. Let us illustrate this by the distribution of tangential stresses in the contact plane. The tangential stress distribution can be easily calculated from the linear force density 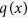 in the one-dimensional MDR-model by applying the integral transformation^11^


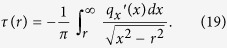


**Figure 4:**
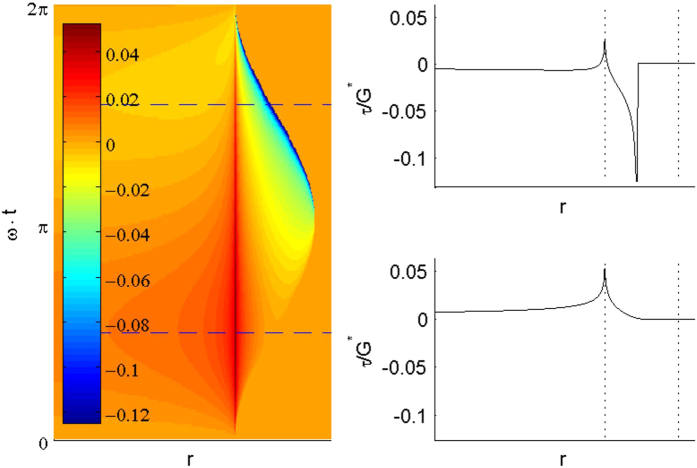
Color map of the distribution of tangential stress as function of radius *r* (horizontal axis) and time (vertical axis) over one period of the oscillation 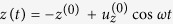
 and 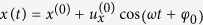 with the phase shift *φ*_0_=*π*/2. At the beginning of the motion, a positive singularity appears at the initial boundary of the contact and remains at this point during the whole oscillation period (right lower subplot.) No energy dissipation is associated with this non-moving singularity. At the moment of reversal of the indentation movement (start of the “pulling” phase) a square-root-singularity appears at the right boundary of the contact and moves subsequently to the left, together with the shrinking contact region (right upper sub-plot.) At the same time, irreversible energy dissipation takes place. The right subplots correspond to the times shown in the color map with horizontal dashed lines. In the sub-plots, the maximum and the minimum extent of the contact region during an oscillation period are shown with dotted lines.

The tangential stress as a function of coordinate and time is shown in Fig. 4 as a color map. Of interest is the range of coordinates where the indenter is in contact only over some part of the oscillation period. In this range, one can see two maxima of the stress: the first one is located at the left boundary of the range. A detailed analysis shows that this is a logarithmic singularity, which is “pulsating” but not moving spatially. The second singularity is located at the right boundary of the contact; it develops and persists during the phase of the oscillation when the indenter is “pulled away”. This is a “square root singularity”, which is moving spatially. Movement of this singularity leads to infinitely rapid movements in the medium even if the indenter is moving quasi-statically. The existence of a singularity of tangential stress distribution is a general property of any contact configuration with infinite friction^27^, which is also confirmed by our numerical analysis.

In the realistic case of finite coefficient of friction, there will be no singularity of tangential stress due to the appearance of a slip region at the boundary of the contact area. Let us discuss the process of energy dissipation in this case. Note that the method of dimensionality reduction is also applicable to the superimposed normal and tangential contact in the presence of a finite coefficient of friction^11^. The process of dissipation of the elastic energy of the “border springs” of the equivalent elastic foundation described at the beginning of the paper will now occur not instantly at the moment of loss of contact but continuously during a finite interval shortly before loss of contact, so that at the moment of final separation the springs will be completely un-stressed. However, if the interval of stress relaxation is small enough, the amount of energy loss will be practically independent of whether it was lost instantly or during a very short time (or displacement) interval. This amount is equal to the elastic energy stored in the border springs before the start of the relaxation process and does not depend on the details of the dissipation mechanism. The same amount of energy would be dissipated if the coefficient of friction were infinite but the material had viscoelastic properties. The application of the MDR in this case requires the replacement of the springs of the elastic foundation by corresponding rheological elements^11^. Let us discuss the simplest case of the Kelvin body. In this case, the elements of the linear viscoelastic foundation will consist of springs connected in parallel to linear dampers. During the superimposed normal and tangential oscillations, such an element will come into contact and will be dragged tangentially exactly as described in the case of purely elastic elements at the beginning of the paper. During this process, elastic energy will be stored in the spring. At the moment when the normal pressure becomes zero, the element starts relaxing. If the relaxation time of the viscoelastic material is much smaller than the period of oscillations, then practically the whole elastic energy will be dissipated. Its amount again, is given by Eq. (8) or for the whole contact by (12). Thus, the dissipative contribution described in the paper will be, in the case of a viscoelastic material, the same as in the elastic case provided the relaxation time of the viscoelastic materials is much smaller than the period of the oscillation (so that during the non-contact time the material can really almost completely relax). The most important feature of the considered dissipation mechanism is that the amount of the dissipated energy is completely independent of the particular dissipation mechanism, be it microslip or internal dissipation in the material. Its basic mechanism is that the pre-stressed spring becomes unstressed (relaxed) due to normal movement. The term “relaxation damping” reflects this physical mechanism of energy loss.

## Discussion

In conclusion, the effect of relaxation damping was discussed using the example of axis-symmetric elastic bodies with infinite friction in the contact area. The discussion was generalized to bodies with arbitrary surface topography, in particular multi-contact systems and contact of bodies with rough surfaces. We have shown that a superposition of normal and tangential oscillation (both with equal and different frequencies) leads to a specific damping, which we call “relaxation damping”. The damping is proportional to the amplitude of the normal oscillations and to the square of the amplitude of the tangential oscillations. For nominally flat rough surfaces, it is also proportional to the applied normal force. The assumption of the infinite coefficient of friction was made only to study the effect in the pure. However, all results are directly applicable to systems with a finite coefficient of friction provided that the changes in the radius of the stick region are much smaller than those due to changing indentation. To show this, let us compare the dissipated energy per cycle due to purely tangential vibration (“Mindlin contribution”^1^), 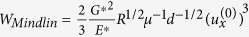 (which can also be written as 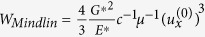) with the energy lost (11) due to relaxation damping. The relaxation damping exceeds the Mindlin damping if 
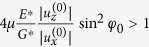
. Note that the left-hand side of this inequality is proportional to the ratio of the changes in the contact radius due to purely normal and purely tangential oscillation.

The above comparison provides an impression of the relative importance of the Mindlin damping and the relaxation damping. As E^*^/G^*^ and sin^2^*φ*_0_ typically have the order of magnitude of unity, the relative importance of the “Mindlin damping” and the relaxation damping is given by the factor 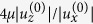. For example, for the coefficient of friction of *μ*=1/4 the relative contribution will be given just by the ratio of the normal and tangential oscillation, 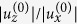. For a typical contact in a system subjected to vibrations, it is common for normal and tangential oscillations to have the same order of magnitude. This means that the relaxation damping for a “typical system” has the same order of magnitude as frictional dissipation.

Let us stress that the present paper is based on the assumption that the amplitude of the tangential oscillation is much smaller than the radius of the contact. While this condition is met on the macroscopic scale (through the assumption of small oscillation amplitudes), it can be easily violated on the scale of microcontacts^28^. This may pose some restrictions to the applicability of the equations (12) and (18) to contacts of rough surfaces. Another restriction is due to the assumption of perfect elasticity. We neglected any kinetic processes, such as creep of micro-contacts^29^, which lead to deviations from the theory already in the case of pure tangential loading and surely have to be considered in the general case as well.

Our analysis shows that application of normal oscillations will significantly change the damping behaviour of tangential movement in a system with friction. This may be used for designing and tuning structural damping of systems with frictional contacts. Further, the effect of the relaxation damping may account for the well-known effect of suppression of frictional instabilities by application of ultrasonic oscillations, which was studied both theoretically^30^ and experimentally^31^.

## Methods

In the theoretical part, we use the Method of Dimensionality Reduction (MDR) in contact mechanics^11^. Within the usual assumptions of contact mechanics, the MDR has been proven rigorously for normal and tangential contacts of simple (axially symmetric) surfaces.

Additional verification and extension to non-axisymmetric indenters is done using the three-dimensional Boundary Element Method (BEM)^25^. We use an implementation of the BEM that is based on the Fast Fourier Transform, and was developed by one of the authors (R.P.)

^*^Let us briefly sketch the reasons for this supposed generality. Consider a contact of an *arbitrarily* shaped rigid indenter with an elastic half-space and assume the decoupling of the normal and tangential problems. The normal force *F*_*N*_ will then depend only on the indentation depth *d*, that is *F*_*N*_=*F*_*N*_(*d*). Let us define the incremental normal contact stiffness *k*_*z*_(*d*) and the incremental tangential stiffness *k*_*x*_(*d*). Now we simultaneously change the indentation by d*d* and the tangential displacement by d*x* and calculate the incremental changes of the normal and tangential forces: d*F*_*N*_=*k*_*z*_(*d*)d*d*, d*F*_*x*_=*k*_*x*_(*d*)d*x*. The ratio of these increments is equal to d*F*_*N*_/d*F*_*x*_=(*k*_*z*_(*d*)/*k*_*x*_(*d*))(d*d*/d*x*). For all axis-symmetric contacts, the ratio of the normal and tangential stiffness is constant and equal to *k*_*z*_(*d*)/*k*_*x*_(*d*)=E^*^/G^*^, see^15^. Indeed, the integral relations connecting the normal and tangential displacements in the origin of coordinates (*x*=*y*=0) with normal and tangential stresses read


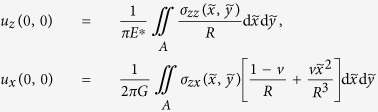


with 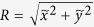. In the axis-symmetric case, the second term in the previous equation can be averaged over the polar angle in the contact area providing


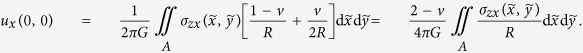


The same stress distributions in normal and tangential direction will thus produce displacements whose ratio is *u*_*z*_(0,0)/*u*_*x*_(0,0)=4*πG*/((2−v)E^*^)=G^*^/E^*^, which means that the stiffness ratio is E^*^/G^*^. This result happens to be extremely robust and is valid in good approximation not only for axis-symmetric contacts. For example, in^22^, it was shown theoretically and numerically that the ratio of the normal and tangential stiffness remains the same for arbitrary randomly rough surfaces. If we assume that this is valid for any contact configuration, then for the ratio of forces we get d*F*_*N*_/d*d*=(E^*^/G^*^)*dF*_*x*_/d*x* which means that the *tangential reaction of any contact is uniquely determined by its normal reaction*. In other words, if for two contact systems the normal reaction *F*_*N*_(*d*) is identical, then the tangential reaction will also be identical. In the papers^23^,^24^ this has been confirmed by numerical simulation of contacts of rough surfaces with arbitrary coefficient of friction. This further means that any arbitrary contact satisfying the conditions of decoupling of the normal and tangential contact behaves in the same way in terms of displacement and forces as an equivalent single-contact axisymmetric system having the same normal reaction. From this it follows that all properties that depend solely on the force-displacement reactions of the system will be identical for all contacts having the same dependence of the normal force on indentation. This provides further support to the generality of the equations (12) and (17).

## Additional Information

**How to cite this article**: Popov, M. *et al.* Relaxation damping in oscillating contacts. *Sci. Rep.* **5**, 16189; doi: 10.1038/srep16189 (2015).
